# Alterations in T and B Cell Receptor Repertoires Patterns in Patients With IL10 Signaling Defects and History of Infantile-Onset IBD

**DOI:** 10.3389/fimmu.2020.00109

**Published:** 2020-02-06

**Authors:** Lael Werner, Yu Nee Lee, Erez Rechavi, Atar Lev, Baruch Yerushalmi, Galina Ling, Neil Shah, Holm H. Uhlig, Batia Weiss, Raz Somech, Scott B. Snapper, Dror S. Shouval

**Affiliations:** ^1^Pediatric Gastroenterology Unit, Edmond and Lily Safra Children's Hospital, Sheba Medical Centre, Ramat Gan, Israel; ^2^Sackler Faculty of Medicine, Tel Aviv University, Tel Aviv, Israel; ^3^Pediatric Department A, Edmond and Lily Safra Children's Hospital, Sheba Medical Centre, Ramat Gan, Israel; ^4^Immunology Service, Edmond and Lily Safra Children's Hospital, Sheba Medical Centre, Ramat Gan, Israel; ^5^Jeffrey Modell Foundation Center, Edmond and Lily Safra Children's Hospital, Sheba Medical Centre, Ramat Gan, Israel; ^6^Pediatric Gastroenterology Unit, Soroka University Medical Center, Ben Gurion University of the Negev, Be'er Sheva, Israel; ^7^Department of Gastroenterology, Great Ormond Street Hospital, London, United Kingdom; ^8^Translational Gastroenterology Unit, Nuffield Department of Experimental Medicine, John Radcliffe Hospital, University of Oxford, Oxford, United Kingdom; ^9^Department of Pediatrics, University of Oxford, Oxford, United Kingdom; ^10^NIHR Oxford Biomedical Research Centre, University of Oxford, Oxford, United Kingdom; ^11^Division of Gastroenterology, Hepatology and Nutrition, Boston Children's Hospital, Boston, MA, United States; ^12^Harvard Medical School, Boston, MA, United States

**Keywords:** IBD, VEOIBD, NGS, T cell receptor repertoire, B cell receptor repertoire, IL10, IL10R, adaptive immunity

## Abstract

Patients with loss-of-function mutations in IL10 or IL10 receptor (IL10R) genes develop severe, medical-refractory, infantile-onset inflammatory bowel disease (IBD). We have previously reported significant alterations in innate and adaptive immune responses in these patients. Next generation sequencing platforms enable a comprehensive assessment of T cell receptor (TCR) and B cell receptor (BCR) repertoire patterns. We aimed to characterize TCR and BCR features in peripheral blood of patients with deleterious IL10 signaling defects. DNA was isolated from blood of seven patients with IL10R mutations and one with an IL10 mutation, along with eight controls, and subjected to next generation sequencing of *TRB* and *IgH* loci. A significant increase in clonality was observed in both TCR and BCR repertoires in circulating lymphocytes of IL10/IL10R-deficient patients, but to a much greater extent in T cells. Furthermore, short CDR3β length and altered hydrophobicity were demonstrated in T cells of patients, but not in B cells, secondary to lower rates of insertions of nucleotides, but not deletions, at the V-, D-, or J-junctions. We were unable to observe specific T or B clones that were limited only to the patients or among controls. Moreover, the expanded T cells clones were unique to each patient. In conclusion, next generation sequencing of the TCR and BCR is a powerful tool for characterizing the adaptive immune cell phenotype and function in immune-mediated disorders. The oligoclonality observed among IL10/IL10R-deficient patients may suggest specialization of unique clones that likely have a role in mediating tissue damage. Nevertheless, the lack of shared clones between patients provides another piece of evidence that the adaptive immune response in IBD is not triggered against common antigens. Additional studies are required to define the specific antigens that interact with the expanded IL10/IL10R-deficient clones.

## Introduction

Inflammatory bowel disease (IBD), such as Crohn's disease (CD), and ulcerative colitis (UC), are chronic, relapsing-remitting inflammatory disorders of the gastrointestinal tract. These diseases develop in genetically susceptible subjects due to a dysregulated immune response to microbial dysbiosis and/or environmental changes (1). Most cases of IBD present between the ages of 20–40 years, and in up to 20% disease manifests in childhood, mainly in adolescent years. However, in 1–2% these disorders present in the first 5–6 years of life, defined as very early-onset IBD (VEO-IBD) (2), whereas infantile-onset IBD is defined when manifestations develop before the age of 2 years.

Extensive use of advanced sequencing platforms in the last decade has facilitated identification of more than 50 different monogenic disorders that directly cause IBD (3). Most of these patients develop IBD in the first years of life (3). Patients with deleterious mutations in IL10 and the IL10 receptor (IL10R) were first reported in 2009 and 2010 (4–6). These patients develop colitis and perianal disease in the first months of life, along with arthritis and folliculitis. In addition, they possess an increased risk of developing diffuse large B cell lymphoma (7, 8). The gastrointestinal disease is severe and refractory to multiple immunosuppressive medications, including steroids, thiopurines, and TNFα antagonists (5, 9). We have reported in two patients that anakinra, an IL1 receptor antagonist, was effective in ameliorating colitis (10) and was used as a bridge to hematopoietic stem cell transplantation (HSCT), which is the treatment of choice in these conditions (5, 9).

Our previous studies have elucidated important roles of IL10 in regulating different immune responses (11). IL10 is a key anti-inflammatory cytokine secreted by immune and non-immune cells. It signals through the IL10 receptor (IL10R), which is a heterotetramer composed of two sub-units of IL10Ra (IL10R1 in humans) that bind IL10 and two sub-units of IL10Rb (IL10R2) that stabilize this complex. Ligation of IL10 to its receptor leads to phosphorylation of STAT3, which translocates to the nucleus and initiates an anti-inflammatory transcriptional program (11). In innate cells, we have shown that IL10 regulates macrophage development and canonical and non-canonical inflammasome-mediated IL1β production (10, 12). In T cells we demonstrated that loss of IL10R-dependent signals enhance a T_H_17 phenotype, but is not required for regulatory T cells (Tregs)-mediated suppression (13). There is paucity of data on IL10R regulation of B cell responses in both mice and humans.

T and B cells express unique antigen-specific receptors that are formed following a complex rearrangement of genetic segments (14). This process involves recombination of V, D, and J gene segments, accompanied by a deletion as well as insertion of random nucleotides, generating millions of T and B cell clones that bind unique antigens. Next generation sequencing (NGS) platforms allow detailed assessment of the T and B cell receptor (TCR and BCR) repertoires at the nucleotide level (15, 16). Such a strategy permits an in-depth overview of adaptive immune cell composition and allows determining whether specific diseases are associated with expansion of distinct clones. By applying NGS on the *TRB* region on rectal biopsies, we have recently reported that patients with UC demonstrate oligoclonal expansion of specific clonotypes in the rectum, while the blood was relatively polyclonal (17). NGS of the *TRB* and *IgH* loci has also been used to characterize adaptive immune function in other immune-mediated disorders, such as juvenile idiopathic arthritis (18, 19), malignancies (20), and primary immunodeficiencies (21, 22).

Given the unique clinical presentation of patients with deleterious IL10/IL10R mutations, and the known alterations in adaptive immune function in these patients, we aimed to characterize T and B cell repertoire patterns in patients with IL10 signaling defects. We hypothesized that these patients possess unique T and B cell repertoire patterns.

## Methods

### DNA Isolation

The study was approved at each site by the local ethics committees. Informed written consent was obtained from each participating subject. Genomic DNA was extracted from blood using a commercially available kit (Wizard kit, Promega), according to the manufacturer's instructions. Up to 2 μg DNA were used for TRB and IgH repertoire library generation by Adaptive Biotechnologies platform (Seattle, USA), with a minimum input of 0.7 μg DNA. Amplified sequences were subjected to high-throughput sequencing using Illumina technology. To ensure equal depth of sequencing among the different samples, we used the Survey level (up to 500,000 reads per sample). Further details for the pipeline utilized for corrections of PCR clustering and sequencing errors can be found in previous reports (15, 23).

### TREC and KREC Analysis

T-cell receptor excision circle (TREC) and kappa-deleting recombination excision circle (KREC) copy numbers were determined as described previously (24). Briefly, 50 ng genomic DNA per sample was used in quantitative real-time polymerase chain reaction (qRT–PCR). A standard curve was constructed using serial dilutions containing 10^3^-10^6^ copies of a known TREC or KREC plasmid. Patient and control samples were tested in triplicate, and the number of TREC or KREC copies in a given sample were calculated by comparing the obtained cycle threshold (Ct) value of the sample to the standard curve using a quantification algorithm.

### TCRβ and IgH Repertoire Analysis

ImmunoSeq software was used for determination of productive clonality, clonal sharing, CDR3β, and CDR3H length, and percent productiveness. Percentage of T cells was calculated using housekeeping genes as controls to measure the total number of nucleated cells. Clonality is calculated as 1-normalized Shannon's entropy. This measures how evenly receptor sequences are distributed amongst a set of T or B cells, with values ranging from 0 to 1. Values near 1 represent samples with one or a few predominant clones (monoclonal or oligoclonal samples) dominating the observed repertoire. Clonality values near 0 represent polyclonal samples. Shannon's H, which measures the overall diversity in a given population, and takes into account the number of unique sequences (richness of the repertoire) and how evenly the sequences are distributed, was calculated using the following formulas:

$$\begin{array}{l} \text{Shannon′s H}=-\sum_{i=1}^{R}\;p_{i}\text{ In }p_{i}\; \end{array}$$

R = Total templates

i = Unique rearrangements

p_i_ = Proportion of the total sequences belonging to the “i”th unique rearrangement

Graphical presentation of the repertoire was presented using hierarchical tree maps using the Treemap software (www.treemap.com). Hydrophobicity scale was determined using a modified version of the Kyte-Doolittle index (25) of average hydrophobicity as previously described (26). Briefly, for each unique CDR, the amino acids composition at consequential positions was determined and value of hydrophobicity assigned. Average hydrophobicity at each position was calculated for all CDRs. Our calculation examined clones with length of 14 amino acids for T cells, and 18 amino acids for B cells; as these were found to be the average lengths of the CDR3β and CDR3H regions, respectively. Unless stated otherwise, all analyses were performed solely on rearrangements (defined as a single, unique nucleotide sequence) which were productive. Analysis for CDR3 length and hydrophobicity were performed on unique rearrangements (1 rearrangement per clone), in order to exclude effects of the expanded clones.

### Statistical Analysis

Values are expressed as mean ± standard error of the mean (SEM). Unpaired Student's *t*-test was used to test for statistical significance. Significance was determined if *P* ≤ 0.05 (with *P*-value summaries as: ^*^P ≤ 0.05, ^**^P ≤ 0.01, and ^***^P ≤ 0.001). For differential *V*-, *D*-, and *J*- gene usage, Bonferoni's adjustment for multiple comparisons was performed.

## Results

### Clinical and Genetic Characteristics of the Patients

Genomic DNA was isolated from peripheral blood of seven IL10R-deficient patients and one IL10-deficient patient. All patients presented with severe colitis and perianal disease within the first months of life, expect for Patient 6 who had an atypical course and developed associated symptoms at age 3 years (Table 1). All patients were refractory to multiple immunosuppressive medications (data not shown). One patient (P5) developed diffuse large B cell lymphoma at the age of 19 years, 9 years before he was identified with IL10R deficiency. Deleterious variants in the *IL10RA, IL10RB*, or *IL10* were identified in all patients, and presented in Table 1. As controls we obtained blood samples from eight pediatric patients referred for evaluation of abdominal pain or diarrhea, but without a diagnosis of IBD, celiac disease, or another immune-mediated disorder. Although the mean age at the time of repertoire analysis was not statistically different between the groups (10.5 ± 1.9 vs. 7.0 ± 3.3 years among controls and patients, respectively; *P* = 0.38), four patients were 2 years of age or younger, compared to none among controls. Basic lymphocyte immunophenotyping for seven patients, performed as part of clinical care, is presented in Table 2.

**Table 1:** Clinical and genetic characteristics of the patients.

**Pt**	**Age of symptom's onset (months)**	**Current age (years)**	**Gender**	**Mutation**	**Clinical presentation**
1	<3	2	F	*IL10RA* c.2T .G; .Met1Arg	Colitis, perianal abscesses
2	<3	13	F	*IL10RB* c.50-2A .T	Colitis, perianal abscesses, bronchiectasis
3	3	2	M	*IL10RB* c.*C52T; 3′ UTR	Colitis, perianal fistulas, arthritis, folliculitis
4	<6	1.5	M	*IL10RA* c.537G .A	Colitis, perianal fistulas
5	<2	28	M	*IL10RA* c.493C .T; *IL10RA* c.689 10G . A	Colitis, enterocutaneous fistulas, history of DLBCL
6	36	5	M	*IL10RA* (c.301C>T)	Colitis, perianal fistulas, arthritis, cortical blindness, diabetes insipidus
7	4	<1	F	*IL10RB* p.Trp18fsX29	Colitis, perianal disease
8	4	<1	M	*IL10* p.Gly113Arg	Colitis, perianal disease

*DLBLC, diffuse Large B cell lymphoma; F, female; M, male; Pt, patient*.

**Table 2:** Lymphocyte Immunophenotyping among patients with IL10/IL10R mutations.

	**P1**	**P2**	**P3**	**P4**	**P6**	**P7**	**P8**
WBC (cells/mm^3^)	NA	NA	20,000	6,350	4,700	6,640	NA
% Lymphocytes	NA	NA	19%	41%	9%	33%	NA
Lymphocytes (cells/mm^3^)	7,814	1,893	3,800	2,572	404	2,210	NA
CD3 (%)	62	82	71	84	76	70	71
CD3 (cells/mm^3^)	4,845	1,552	2,698	2,160	307	1,550	NA
CD4 (%)	23	42	30	42	33	39	47
CD4 (cells/mm^3^)	1,813	793	1,140	1,080	133	860	NA
CD8 (%)	36	33	43	38	38	26	23
CD8 (cells/mm^3^)	2,818	628	1,634	977	154	570	NA
CD4/CD8 ratio	0.6	1.3	0.7	1.1	0.9	1.5	2.0
CD19/20 (%)	27	6	15	10	7	23	21
CD19/20 (cells/mm^3^)	2,078	109	570	257	28	510	NA

*NA, not available; WBC, while blood cell count*.

### TREC and KREC Analysis in IL10/IL10R Deficient Patients

TREC are small circular DNA segments that are created in T cells during TCR rearrangement in the thymus, and thus can reflect thymic output (27). Similarly, KREC reflects B cell output from the bone marrow. TREC values were significantly lower in IL10/IL10R-deficient patients vs. controls (436 ± 158 vs. 2,302 ± 412 copies/50 ng DNA; *P* ≤ 0.01, Supplementary Figure 1A). Specifically, two of the older subjects (aged 13 and 30 years) in the patient's group exhibited low levels (64 and 149 copies/50 ng DNA), which may be in part to decreasing TREC values with age (28). Nevertheless, even when these two samples were excluded from analysis, TREC levels were significantly lower in patients in comparison with controls (data not shown). In B cells, KREC levels were comparable between groups (Supplementary Figure 1B). These data may suggest a defect in thymic output of T cells, but not of bone-marrow derived B cells, in patients with IL10 signaling defects.

### Alterations of TCR Repertoire Features in Blood of Patients With IL10/IL10R Mutations

**Figure 1:**
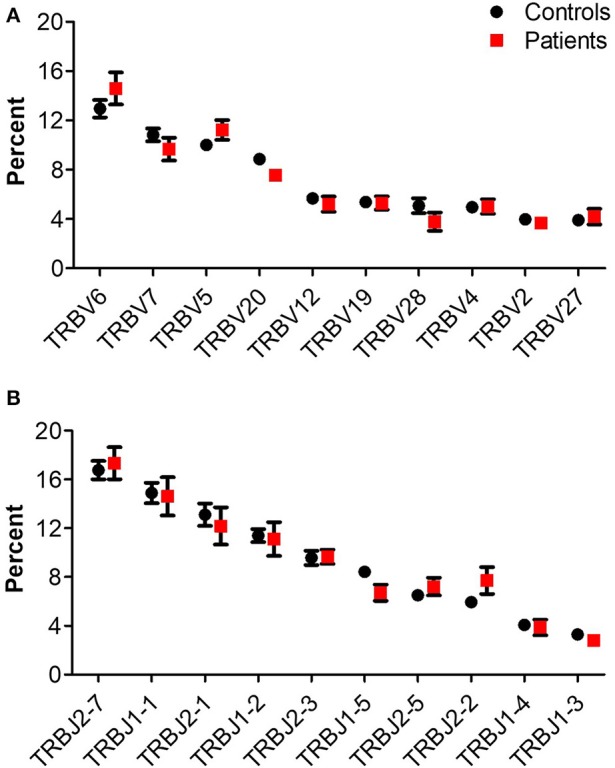
*TRB* gene usage in patients with IL10/IL10R defects and controls. Comparison of usage of the 10 most frequently used **(A)** *TRBV* and **(B)** *TRBJ* genes between control and patient groups.

The number of total TCR rearrangements among patients and controls was comparable (44,852 ± 7,866 vs. 63,100 ± 20,893; *P* = 0.43); as were the unique rearrangements (35,439 ± 6,184 vs. 50,578 ± 16,635; *P* = 0, see Table 3). The calculated percentage of circulating T cells, based on NGS data, was also similar between groups (29.5 ± 5.3% vs. 29.2 ± 7.5% among patients and controls, respectively; *P* = 0.98). Next, we examined differential *TRBV* and *TRBJ* gene usage among unique rearrangements of IL10/IL10R-deficient patients, in comparison to controls. *TRBV* and *TRBJ* gene usage was similar between the groups, and specific patterns of gene usage could not be observed (Figures 1A,B).

**Table 3:** Sample overview of participating subjects.

	**T cells**			**B cells**		
	**Unique rearrangements**	**Total rearrangements**	**% T Cells**	**Unique rearrangements**	**Total rearrangements**	**% B cells**
C1	7,497	9,148	2.8	22,656	27,143	5.5
C2	6,212	7,922	6.6	24,434	29,748	7.9
C3	78,144	97,256	43.9	15,475	18,792	9.9
C4	14,355	17,108	5.4	61,417	74,230	20.4
C5	129,360	160,631	50.6	48,932	60,651	11.4
C6	101,785	129,980	41.5	
C7	23,687	29,594	30.6	
C8	43,581	53,159	52.3	
P1	35,388	43,495	33.9	94,317	112,512	52.9
P2	50,675	61,800	36.5	3,957	4,671	3.6
P3	52,487	68,677	29.3	
P4	35,109	45,854	53.4	13,539	16,471	5.6
P5	15,974	19,043	7.1	320	396	0.2
P6	57,079	71,993	24.6	
P7	28,161	36,882	38.8	12,985	16,038	14.0
P8	8,635	11,069	12.0	876	1,114	0.5

**Figure 2:**
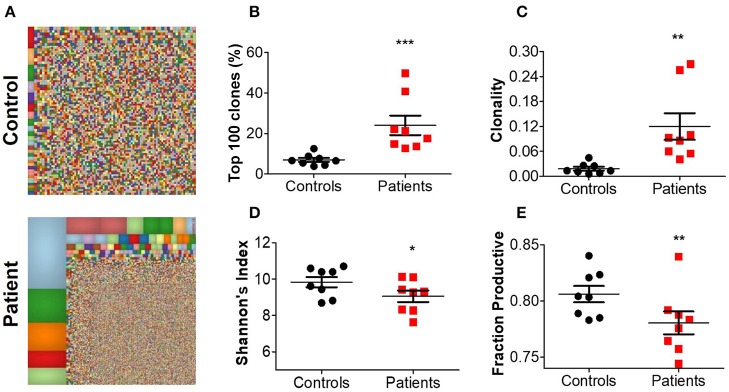
Aberrant TCR repertoire features in blood from patients with IL10 signaling defects. **(A)** Representative Treemaps of entire TCR repertoire in a control (C1) and an IL10R-deficient patient (P2). **(B)** Cumulative percentage of the 100 most abundant clones from the total repertoire, **(C)** calculated clonality, **(D)** Shannon's diversity index, and **(E)** fraction productiveness in control and patient groups. Bars represent mean ± SEM.

For a broad overview of the overall repertoire, we performed Treemap analysis. In these images, each square represents a specific amino acid sequence, and the size correlates with its frequency. Marked clonal expansion was observed in patients with IL10 signaling defects, compared with controls (Figure 2A; Supplementary Figure 2). In Table 4 we present the three most expanded clones in each of the patients, and their respective gene family. The cumulative frequency of the top 100 most frequent clones was significantly higher among patients vs. controls (24.2 ± 4.2 vs. 14.1 ± 1.5%; *P* ≤ 0.001, Figure 2B). Furthermore, the calculated clonality was significantly higher in the blood of IL10/IL10R-deficient patients (Figure 2C) and inversely, Shannon's H diversity index was significantly lower (Figure 2D), compared with controls.

**Table 4:** The amino-acid sequence, and corresponding TRBV-TRBJ pair, of the three most expanded T cell clones for each of the patients.

**Subject**	**Amino acid**	**V-J Genes**	**Percent (%)**
P1	CASSLGGGPYEQYV	V5-6 J2-2	5.13
	CASRRGPLATGELFF	V6-5 J2-2	4.32
	CASSLQGREKLFF	V27-1 J1-4	4.07
P2	CASSFRGELFF	V7-7 J2-2	9.82
	CASSFGTGLTEQYF	V6-5 J2-7	4.64
	CASSMTGQVGSPLHF	V19-1 J1-6	3.86
P3	CARSFGSYSNQPQHF	V5-3 J1-5	2.13
	CSVRQNTEAFF	V29-1 J1-1	2.02
	CAWEDWRVLQEQFF	V30-1 J2-1	1.09
P4	CAWSGGTEAFF	V30-1 J1-1	3.37
	CSAIGGAYEQYF	V unresolved J2-7	2.07
	CASSFGPQYNQPQHF	V28-1 J1-5	1.96
P5	CASSPGARTEAFF	V19-1 J1-1	1.52
	CASSSNTGVPTGELFF	V unresolved J2-2	1.24
	CASSLEPSEGYGYTF	V4-2 J1-2	1.00
P6	CASSPPETYEQYF	V18-1 J2-7	4.71
	CASSLALSRGEQFF	V7-9 J2-1	1.09
	CASSKAGAGGEQYF	V10-2 J2-7	0.59
P7	CASTGANTEAFF	V9-1 J1-1	9.02
	CASSWGYEQYF	V28-1 J2-7	0.87
	CASSGMNTEAFF	V9-1 J1-1	0.69
P8	CASSEDFGGADEQFF	V6-1 J2-1	3.07
	CASSLSRDTYNEQFF	V27-1 J2-1	2.58
	CASSESAGRGPYEQYF	V6-1 J2-7	1.91

Next, we assessed the fraction of productive clones in each subject. Since a T-cell undergoes random somatic rearrangement, the outcome can be a non-productive allele (for example, generation of stop codon, or out-of-frame sequence). Calculation of the fraction of productive TCR sequences in each sample showed significantly lower productiveness among patients, compared with controls (78.0 ± 1.0 vs. 80.6 ± 0.7%; *P* ≤ 0.01, Figure 2E). Overall, these results suggest that patients with IL10/IL10R mutations exhibit clonal expansion of specific T cell clones, which may have a role in mediating the clinical phenotype.

**Figure 3:**
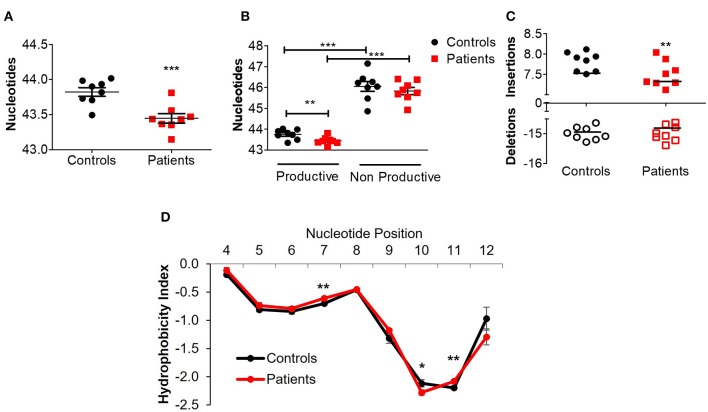
Aberrant TCRβ length and hydrophobicity in T cell lymphocytes of patients with IL10/IL10R defects. **(A)** Mean CDR3β length in circulating lymphocytes of controls and patients. **(B)** Sub-analysis of CDR3β length according to productive and non-productive clones. **(C)** Analysis of the number of nucleotide insertions and deletions in total clones. **(D)** Hydrophobicity index of the CDR3β region. Position signifies successive amino acid location at the CDR3β region. Bars represent mean ± SEM.

Next, we assessed different features of the CDR3β, which is the most variable region of the TCR and thus can affect its antigenic binding affinity. The length of the CDR3β loop itself is important in determining T cell specificity, as longer CDR3β regions have a greater potential for sequence variation and can potentially reach into narrow antigenic pockets (29). The mean CDR3β length in peripheral blood of the patients was significantly lower compared to controls (43.4 ± 0.08 vs. 43.8 ± 0.07 nucleotides, respectively; *P* ≤ 0.001, Figure 3A). This statistically significant difference was maintained at the amino acid level as well (14.48 ± 0.02 vs. 14.59 ± 0.03 in patients and controls, respectively; *P* ≤ 0.01). A sub-analysis revealed similar results when comparing only productive clones (43.4 ± 0.06 vs. 43.8 ± 0.06 nucleotides in patients and controls, respectively; *P* ≤ 0.01), while the length of non-productive clones was comparable between the groups (Figure 3B). These differences in CDR3β length can be partially explained by a significantly lower rate of random insertions of nucleotides, but not deletions, at the *V*-, *D*-, or *J*-junctions, among patients with IL10/IL10R defects (Figure 3C). Interestingly, the length of the non-productive clones was significantly higher compared with the productive clones in both groups (Figure 3B).

Another important biochemical property of the TCR is its hydrophobicity, which could promote development of self-reactive clones (30) and affects antigenic recognition (31), since intrinsic hydrophobicity of specific residues influences binding properties. As shown in Figure 3D, for CDR3β with length of 14 amino acids (the most frequent length), significant differences in hydrophobicity were evident between patients and controls at residue positions 7, 10, and 11. Residues 7 and 11 were significantly more hydrophobic in patients, while residue 10 was significantly less hydrophobic. Examination of hydrophobicity for CDR3β with lengths of either 13 or 15 amino acids, showed no differences between patients and controls (data not shown). Overall, these results may suggest biochemical property differences of the CDR3β in T cells from patients with IL10 signaling defects, which can consequently affect their function.

### BCR Repertoire of IL10/IL10R-Deficient Patients

**Figure 4:**
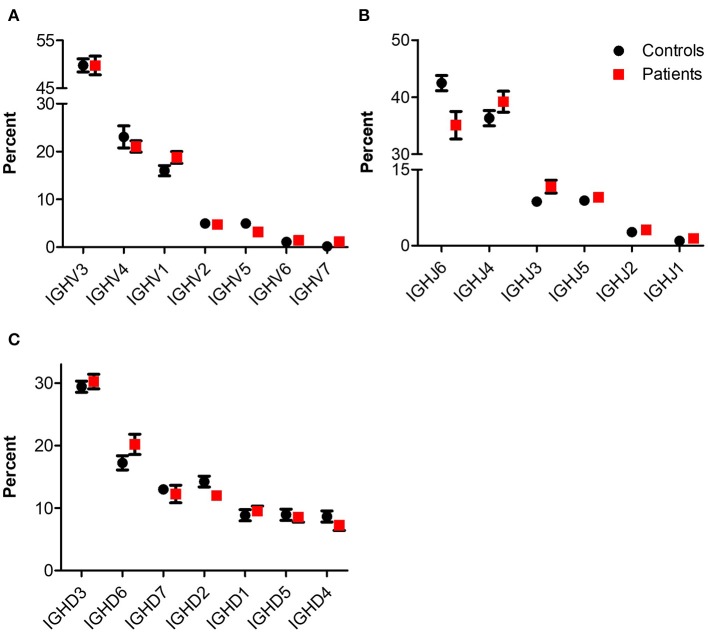
IgH gene usage in patients with IL10/IL10R defects. Comparison of usage of **(A)** *IGHV*, **(B)** *IGHJ*, and **(C)** *IGHD* genes between control and patient groups.

NGS of the IgH genetic segments was performed on blood samples of 6 patients with IL10/IL10R deficiency and five controls. One of the patients (P1), a 2 years old female with an *IL10RA* mutation with severe colitis and perianal disease, but without any signs of lymphoma, displayed a very high percentage of B cells (52.9%) and total rearrangements (112,512). These values were markedly elevated compared with the other patients or controls examined, and therefore were excluded from analysis of frequency and number of rearrangements. The frequency of B cells was slightly decreased in the patient group (4.8 ± 2.5 vs. 11.0 ± 2.5% in patients and controls, respectively; *P* = 0.12). This is also reflected by their lower numbers of total (7,738 ± 3,552 vs. 42,113 ± 10,715 rearrangements among patients and controls, respectively; *P* = 0.02) and unique (6,335 ± 2,896 vs. 34,583 ± 8,764 rearrangements among patients and controls, respectively; *P* = 0.02, see Table 3). We next looked at gene usage among unique rearrangements and found that frequency of *IGHV, IGHJ*, and *IGHD* gene groups were overall similar between groups (Figures 4A–C).

**Figure 5:**
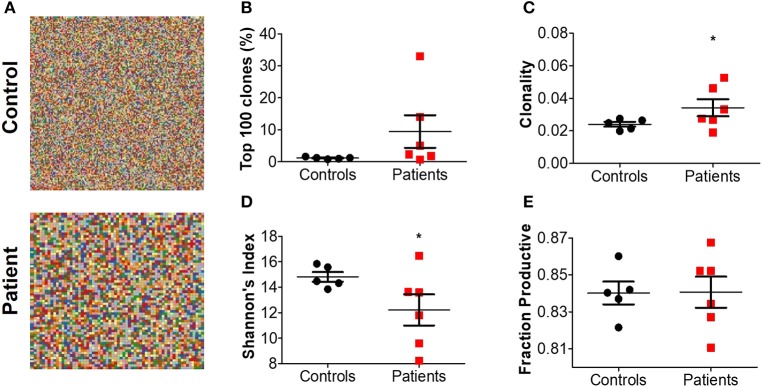
Increased clonality of BCR repertoire in blood from patients with IL10 signaling defects. **(A)** Representative Treemaps of entire BCR repertoire in a representative control (C1) and IL10R-deficient patient (P2). **(B)** Cumulative percentage of the 100 most abundant clones from the total repertoire, **(C)** calculated clonality, **(D)** Shannon's diversity index, and **(E)** fraction productiveness in control and patient groups. Bars represent mean ± SEM.

**Figure 6:**
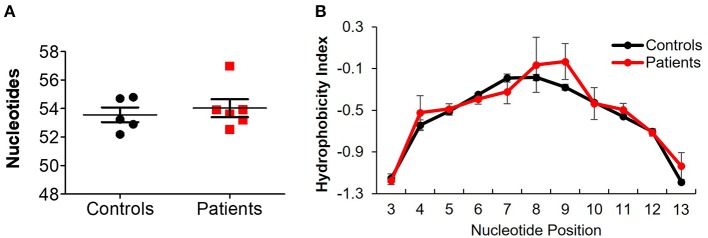
Comparable IgH length and hydrophobicity between controls and IL10/IL10R-deficient patients. **(A)** CDR3H length in blood of patients and controls. **(B)** Hydrophobicity index of the CDR3H region. Position signifies successive amino acid location at the CDR3β region. Bars represent mean ± SEM.

Treemap images demonstrated the lower number of rearrangements among patients (Figure 5A; Supplementary Figure 3). Comparable cumulative frequencies of the top 100 most common clones were observed in both groups, despite a trend for a higher frequency among patients (9.5 ± 5.1 vs. 1.1 ± 0.1%. *P* = 0.08; Figure 5B). The calculated clonality was significantly higher (though still much lower than for T cells, Figure 5C), while Shannon's diversity index was significantly lower (Figure 5D), in patients compared with controls. No difference in productiveness was found between the groups (Figure 5E). Last, the mean CDR3H length (Figure 6A) and hydrophobicity of CDR3H with length of 18 amino acids (Figure 6B) were comparable between groups. Collectively, these results suggest that although some changes in IgH repertoire features were observed in patients with IL10/IL10R defects in the blood, these were much less prominent compared to alterations in TCRβ repertoire.

### Degree of Clonal Sharing Between Controls and Between Patients

Our previous studies among patients with UC demonstrated sharing of specific T cell clones in the gut (but not in the blood), although they were also identified in healthy subjects, but to a lesser extent (17). Such sharing may point to unique clones that have an important role in the pathogenesis of this disorder. In contrast to UC patients, in our IL10/IL10R-deficient patient cohort, there were no unique blood-borne T or B cell clones shared exclusively either among the IL10/IL10R-deficient patients or among the controls. Interestingly, the 10 most expanded T cell clones in each IL10/IL10R-deficient patient were unique, and not identified in other patients, or publicly available databases, such as VDJdb. We did identify public T cell clones (most of them directed against viral antigens) shared between controls and patients, although their frequency was very low (Table 5).

**Table 5:** Shared T cell clones between controls and patients.

**Amino acid**	**Antigen**	**Controls**		**Patients**	
		**Contributors**	**Avg %**	**Contributors**	**Avg %**
CASSLGETQYF	CMV	7	0.0075	7	0.0080
CASSLGDTQYF	Unknown	7	0.0072	7	0.0055
CASSSTDTQYF	CMV	7	0.0058	6	0.0030
CASSLGYEQYF	CMV/EBV	6	0.0069	7	0.0520
CASSLEETQYF	Unknown	6	0.0052	7	0.0036
CASSLGVNTEAFF	CMV	6	0.0059	7	0.0077
CASSLQETQYF	Unknown	6	0.0046	7	0.0061
CASSPSYEQYF	EBV	6	0.1387	6	0.0040
CASSLGGNTEAFF	Unknown	6	0.0054	6	0.0059
CASSLGGYEQYF	EBV	6	0.0046	6	0.0054
CASSLNTEAFF	CMV/EBV/Influenza	6	0.0075	6	0.0077
CASSLSTDTQYF	CMV/EBV	6	0.0035	6	0.0072
CASSLGGSNQPQHF	CMV/EBV	6	0.0093	6	0.0032
CASSLGLNTEAFF	Unknown	5	0.0022	6	0.0083
CASSLAGGYEQYF	CMV	5	0.0038	6	0.0067
CASSPGTSYEQYF	Unknown	4	0.0022	6	0.0066
CASSLGGTEAFF	CMV	5	0.0026	6	0.0057
CASSPQGNTEAFF	Unknown	5	0.0025	6	0.0065

*Table displaying clones that are shared between at least 6 controls or 6 patients. CMV, Cytomegalovirus; EBV, Epstein–Barr virus*.

**Figure 7:**
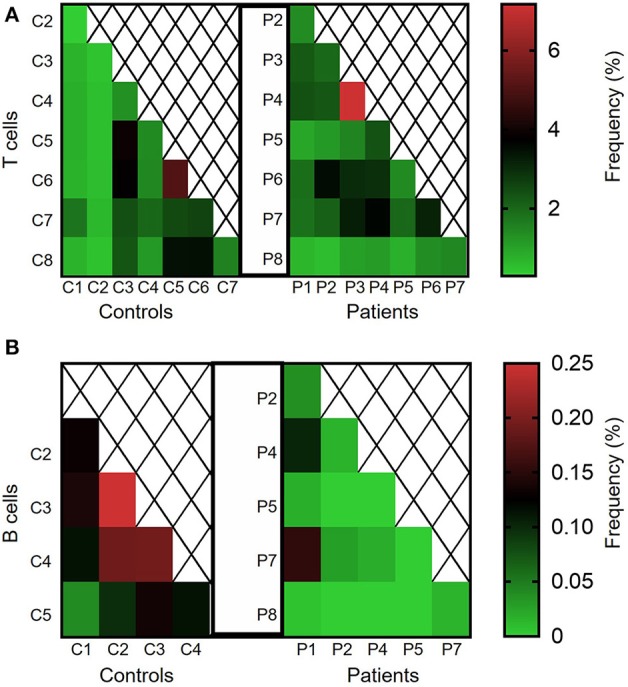
Clonal sharing between controls and between different IL10/IL10R-deficient patients. Degree of clonal sharing is presented in heat maps images, depicting percentage of the **(A)** TCR and **(B)** BCR repertoires shared between any two individuals.

We next calculated the Jaccard index, reflecting degree of clonal sharing between any two individuals (Figures 7A,B). Overall, clonal sharing in the blood was low in both groups, reflecting the heterogenic circulating repertoire. The mean degree of T cell sharing was comparable between groups (2.11 ± 0.25 vs. 1.70 ± 0.24% among patients and controls, respectively; *P* = 0.12, see Figure 7A). However, sharing of clones was lower in B cells of patients vs. controls (0.03 ± 0.01 vs. 0.14 ± 0.02%, *P* ≤ 0.001; Figure 7B). Looking at the nucleotide level, nearly no sharing was identified in both groups for T and B cells (data not shown).

## Discussion

Patients with rare deleterious mutations in the IL10 axis develop a unique infantile-onset form of IBD. As such, we and others have previously characterized various dysregulated immune responses in these patients in both innate and adaptive immunity (10, 12, 32–36). We were able to show that loss of IL10 signaling does not impair the suppressive capacity of Tregs, but leads to enhanced generation of T_H_17 cells and upregulation of IL1-mediated responses (13). Our current study provides another aspect of adaptive immunity in these patients.

Using NGS of the TCRβ and IgH, we were able to deeply characterize T and B cell repertoire features in a relatively large cohort of rare patients with IL10 and IL10R mutations. One of the key findings in our study is marked clonal expansion, mainly in T cells, and to a lesser extent in B cells, in these patients. Upregulation of specific clones is observed in various immune-mediated disorders, primary immunodeficiencies, and malignancies. In our previous study, we have shown that patients with UC also exhibit an oligoclonal TCRβ repertoire; nevertheless, this was observed only in the inflamed colon, but not in the blood (17). This makes our findings on IL10/IL10R-deficient patients even more striking, since in circulating blood there are high frequencies of naïve T cells that likely have a polyclonal distribution. It will be interesting to look in these patients at repertoire features of specific immune subsets, including effector memory, which acquire a TH17 phenotype, and Tregs, which seem to function normally based on our previous observations (13).

A skewed TCRβ repertoire in the blood was previously reported in different primary immunodeficiencies, including Ras-associated lymphoproliferative disease (RALD) (24) and wiskott-Aldrich syndrome (WAS) (18) which demonstrated significant restriction of the TCRβ repertoire. These disorders are associated with recurrent infections and result from mutations in genes critical for normal immune development and function. Patients with deleterious IL10/IL10R mutations are generally not more susceptible to infections, but are still considered to have an immunodeficiency. These abnormal repertoire features, which are not seen in the blood of patients with UC (17) or CD (37), are another reflection of the important role of IL10 in mediating immune responses.

Another altered TCRβ repertoire feature we show in patients with IL10 signaling defects is a significantly shorter CDR3β length and changes in hydrophobicity. Abnormalities in CDR3β length were previously described in primary immunodeficiencies, including in patients with WAS and recombination-activating genes (RAG) mutations, where a correlation between length and severity of phenotype was demonstrated (18, 38). Alterations in distribution of the CDR3β length were also described in autoimmune disorders, including insulin-dependent diabetes mellitus (39), and multiple sclerosis (40). Although the differences in CDR3β appear minor, they might still have functional consequences. For example, in insulin-dependent diabetes mellitus a slight, yet significant decrease in CDR3β length may be associated an increased potential for self-recognition (39). The decreased CDR3β length we describe was demonstrated only in productive clones of patients with IL10 defects (representing >75% of the total repertoire). If shorter CDR3β length were genetically determined in the patients (due to IL10 regulation of thymic selection, for example), we would expect to detect shorter CDRs in the non-productive clones as well, implying that other triggers may be responsible for the reduced CDR3β lengths. Thus, together with the aberrant hydrophobicity of the CDR3β, as hydrophobic residues are thought to form high affinity binding “hotspots” to antigen (41, 42), the characterization of the CDR3β suggests a defect in proper antigen recognition in the TCR repertoire of IL10/IL10R-deficient patients.

Findings of the IgH repertoire features were relatively comparable between groups, despite increased clonality among IL10/IL10R-deficient patients. A trend toward the focus on B cell immune repertoire is important, given the high risk of these patients to develop EBV-negative diffuse large B cell lymphoma (7, 8). The mechanisms leading to lymphoma in these conditions are unclear but might be related to impaired immune surveillance by cytotoxic CD8^+^ T cells (43). One patient in our cohort had a history of lymphoma, 9 years before the diagnosis of IL10R deficiency was made, and blood was obtained for the current study. While we could not identify a specific clone that was markedly upregulated in the blood of these patients, clonal expansion might occur at other sites or at later stages. In the IL10/IL10R-deficient patients lymphoma developed in the intestine, liver and/or spleen (7, 8), and therefore unique B cell signatures might only be identified at that specific sites and not in the blood.

Clonal sharing of specific T and B cell clonotypes was very low in our cohort, both between patients and between controls. In addition, we were unable to identify unique sequences limited to one of the groups (patients or controls). Although our sample size was relatively small, this may suggest that IL10/IL10R deficiency is not triggered by common antigens, and are in line with microbiome studies showing large heterogeneity among IBD patients (44). Nevertheless, sharing of clones might be evident at mucosal surfaces. Additional studies are required to identify specific microbial triggers that stimulate T cells in these patients and lead to clonal expansion.

Our study has several limitations. First, we studied repertoire features of total T and B lymphocytes. While this provides a broad and important overview, it fails to capture changes is specific subsets, especially those that are less frequent, such as Tregs. Analysis of these characteristics in sorted immune populations or in single-cell RNAseq coupled to TCRβ repertoire should provide more specific information. Second, usage of RNA for immune repertoire studies, instead of DNA, would enable detection of somatic hypermutations and isotype switching in B cells. In addition, usage of primers for longer reads within the entire V-region would also allow analysis of somatic hypermutations in B cells. Third, comparisons were performed between patients and controls without IBD or other immune-mediated disorder. While the patients' repertoire features were significantly different from those we reported for UC patients (17), a comparison to a group of patients with a systemic inflammatory phenotype, including those with CD would be valuable, but this was beyond the scope of this study. Another potential limitation is the age discrepancy between control and patient groups, as four of the patients were 2 years old or younger. Britanova et al. reported a decrease in TCR repertoire diversity with age; nevertheless, the youngest subject in that study was 6 years of age, and most participants were adult (45). We have recently reported that the intestinal B and T cell receptor repertoire among infants is diverse, similar to pediatric adolescent subjects (46). Although we did not assess repertoire of circulating blood-borne lymphocytes, we can speculate it is also diverse in the first year of life. Finally, immune repertoire assessment of intestinal samples and analysis of autologous blood-intestinal clones of these IL10/IL10R-deficient patients would be interesting; however, obtaining research biopsies from these young, sick and rare patients is challenging.

In conclusion, we reveal that both the T and B cell receptor repertoires are skewed in patients lacking intact IL10 signaling and are characterized by enhanced clonality, and alterations in several repertoire features, mostly in T cells. Given the severe and unique inflammatory phenotype of these patients, additional studies are required to elucidate the dysfunctional adaptive arm. Last, this study is another proof-of-concept that NGS is a powerful tool for in-depth analysis of adaptive immune function.

## Data Availability Statement

The raw data supporting the conclusions of this article will be made available by the authors, without undue reservation, to any qualified researcher.

## Ethics Statement

The studies involving human participants were reviewed and approved by Sheba Medical Center, Boston Children's Hospital and NIHR Oxford Biomedical Research Center. Written informed consent to participate in this study was provided by the participants' legal guardian/next of kin.

## Author Contributions

LW designed the study, conducted the analysis, and wrote the manuscript. YL, ER, AL, BY, and GL contributed to data analysis. NS, HU, BW, RS, and SS contributed to acquisition of samples. DS designed the study, conducted the analysis, and wrote the manuscript.

### Conflict of Interest

The authors declare that the research was conducted in the absence of any commercial or financial relationships that could be construed as a potential conflict of interest.
