# Supplementary material for: Alterations in T and B Cell Receptor Repertoires Patterns in Patients With IL10 Signaling Defects and History of Infantile-Onset IBD
Source: Front Immunol. 2020 Feb 6;11:109. doi: 10.3389/fimmu.2020.00109 (PMC7017840; doi:10.3389/fimmu.2020.00109)
Supplement: Supplementary file 1 [file Presentation_1.PPTX]

## Slide 1
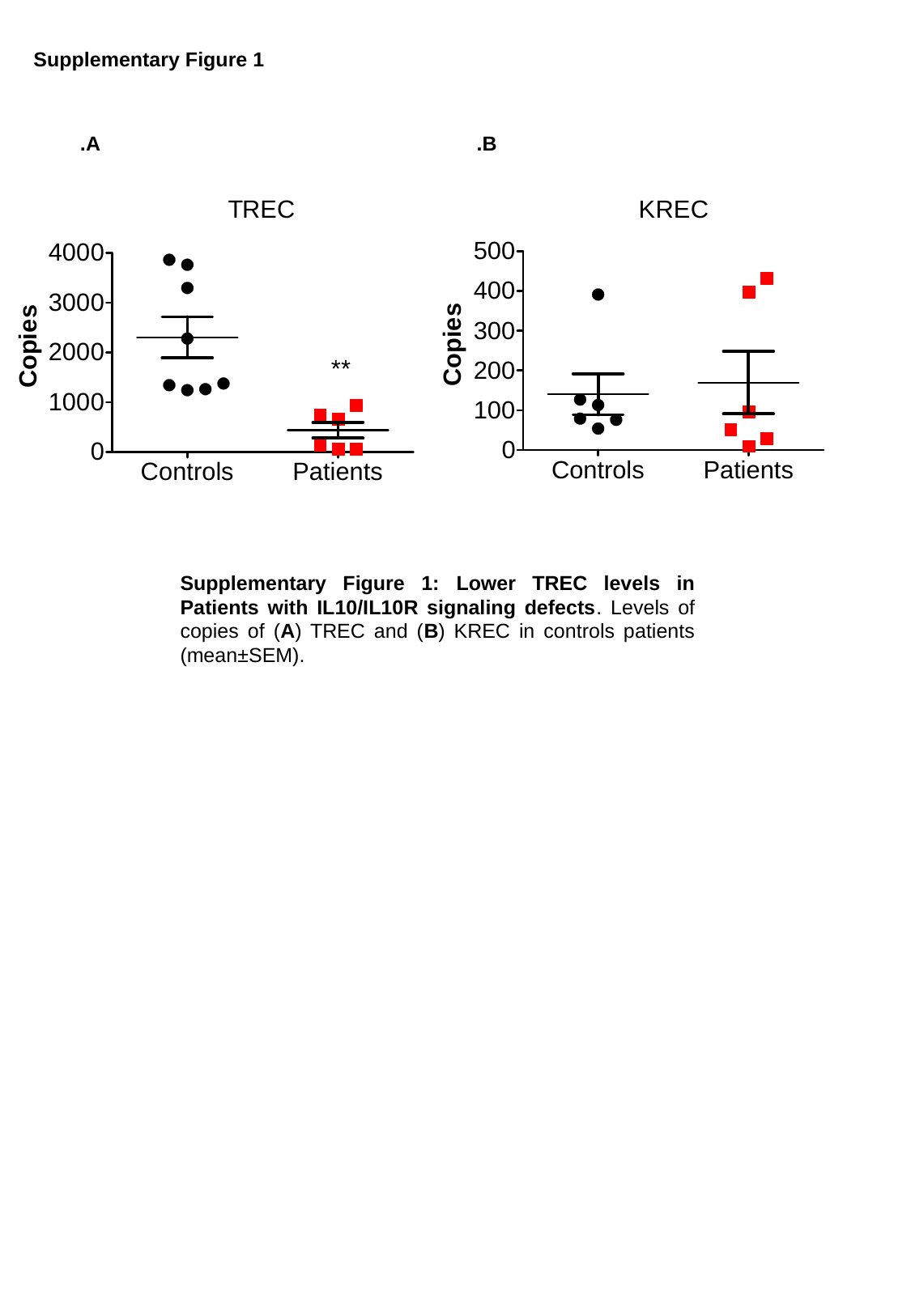

Supplementary Figure 1
A.
B.
Supplementary Figure 1: Lower TREC levels in Patients with IL10/IL10R signaling defects. Levels of copies of (A) TREC and (B) KREC in controls patients (mean±SEM).

## Slide 2
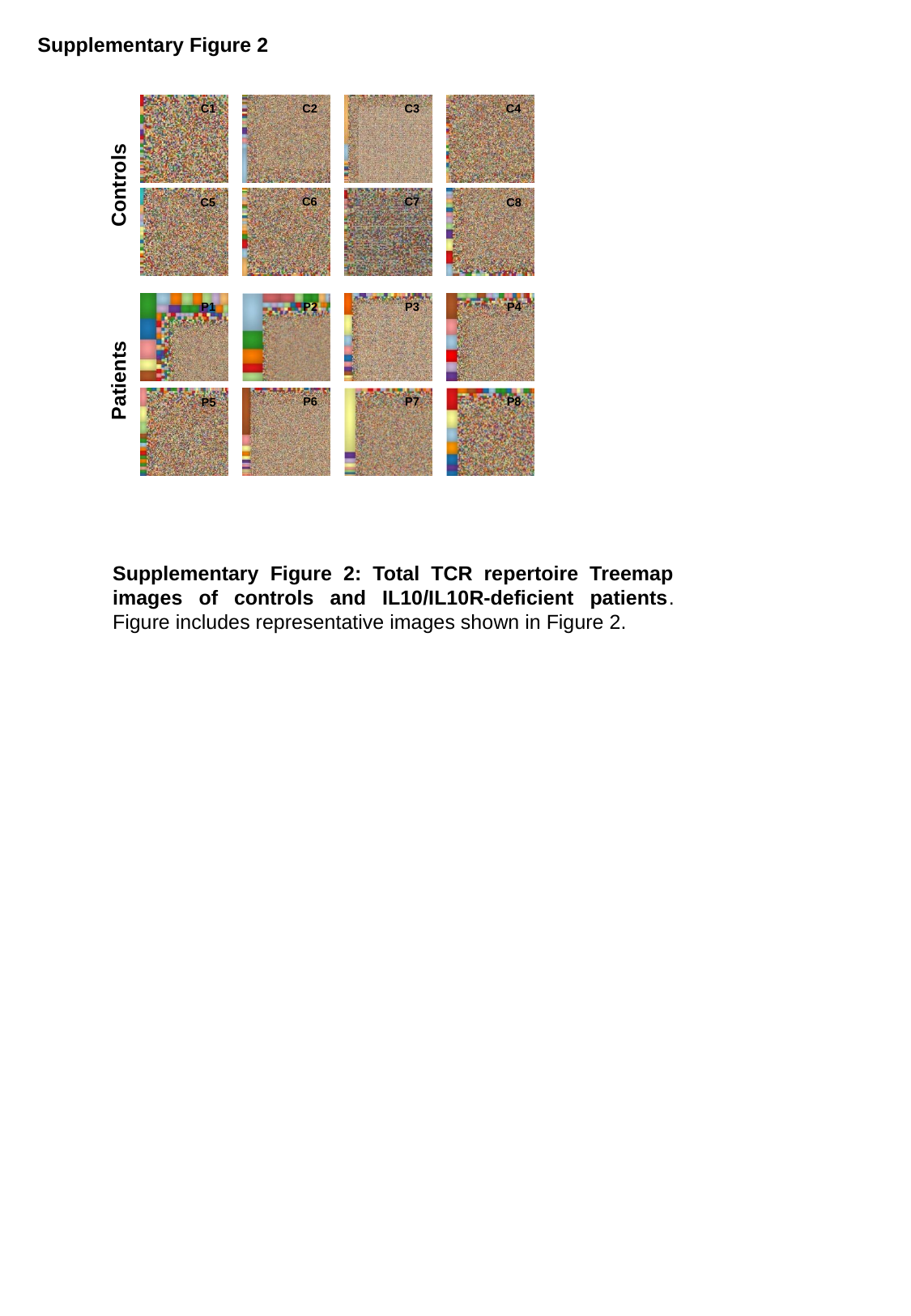

Supplementary Figure 2
C2
C4
C3
C1
Controls
C7
C6
C8
C5
P2
P3
P4
P1
Patients
P7
P6
P8
P5
Supplementary Figure 2: Total TCR repertoire Treemap images of controls and IL10/IL10R-deficient patients. Figure includes representative images shown in Figure 2.

## Slide 3
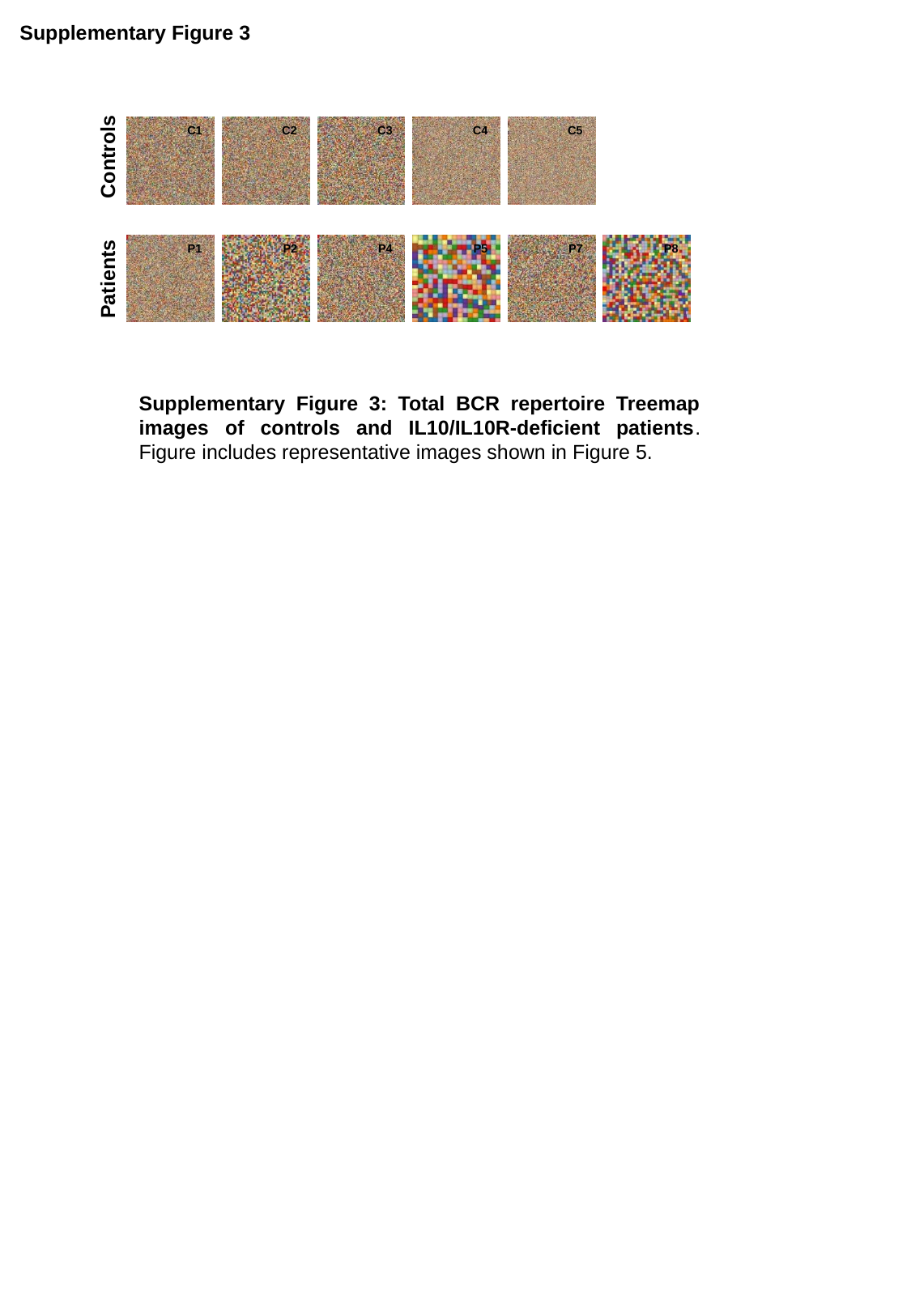

Supplementary Figure 3
C3
C4
C2
C1
C5
Controls
P1
P4
P8
P2
P5
P7
Patients
Supplementary Figure 3: Total BCR repertoire Treemap images of controls and IL10/IL10R-deficient patients. Figure includes representative images shown in Figure 5.
